# Review of Recent Advances in Polylactic Acid/TiO_2_ Composites

**DOI:** 10.3390/ma12223659

**Published:** 2019-11-07

**Authors:** Mosab Kaseem, Kotiba Hamad, Zeeshan Ur Rehman

**Affiliations:** 1 Department of Nanotechnology and Advanced Materials Engineering, Sejong University, Seoul 05006, Korea; 2 School of Advanced Materials Science & Engineering, Sungkyunkwan University, Suwon, Gyeonggi-do 16419, Korea; hamad82@skku.edu; 3 Department of Materials Science and Engineering, Hongik University, Sejong, Jochiwon, Sejong-ro 2639, Korea; zeeshan@hongik.ac.kr

**Keywords:** polylactic acid, titania, mechanical properties, crystallization, photocatalytic activity, antimicrobal properties

## Abstract

Polylactic acid/titanium oxide (PLA/TiO_2_) composites as multifunctional materials have been studied extensively by couple of research groups owing to their outstanding mechanical, thermal, photocatalytic, and antimicrobial properties. This review describes the experimental approaches used to improve the compatibility of PLA/TiO_2_ composites. The mechanical, thermal, photocatalytic, and antimicrobial properties of PLA/TiO_2_ composites are discussed. The potential applications arising from the structural and functional properties of PLA/TiO_2_ composites were also reviewed. Finally, it is concluded that a deep understanding of the impacts of TiO_2_ filler with available improvement approaches in the dispersibility of this filler in the PLA matrix would be the key for the effective usage of PLA/TiO_2_ composites and to expand their suitability with worldwide application requirements.

## 1. Introduction

Titanium oxide (TiO_2_) nanoparticle has gained significant interest owing to its non-toxicity, high functionality toward biomaterials, and high chemical stability [1,2]. It is commonly utilized as a photocatalytic antimicrobial material for packaging applications [3]. It also has excellent antimicrobial activity against a large variety of bacteria [4]. TiO_2_ has three crystallographic phases, anatase, rutile, and brookite which in turn possess different characteristics and different applications. The values of wide band gap were reported to be 3.0 and 3.2 eV for rutile and anatase phases, respectively. Accordingly, the anatase would be more suitable than rutile for biological applications [5]. Owing to the outstanding properties of TiO_2_, a new avenue in the material world is expected to be opened specifically in the field of polymer nanocomposites [6,7,8].

Indeed, polymer nanocomposite materials consisting of TiO_2_ nanoparticles have gained much interest because of their promising properties, finding applications in many fields, such as catalysis, bioengineering, food packaging, biotechnology, biomedical sector [9,10]. Here, it is stressed that the size of particles incorporated into the polymer matrix would greatly affect the properties of the resulting composites. For instance, it was reported that the smaller size of TiO_2_ particles (<30 nm) can exhibit better photocatalytic properties of TiO_2_ in comparison to the larger particles [11].

Polylactic acid (PLA), a biodegradable polyester synthesized from renewable raw materials, is extensively used for medical, packaging, and textile fiber applications [12]. Because of its versatility and the relatively cheap price, PLA became one of the most promising polymers in the last decade. However, the applicability of PLA in some fields could be restricted because of the major drawbacks of PLA including no antimicrobial activity, poor thermal properties and low toughness [12]. Thus, several procedures, such as copolymerization, blending, and inclusion of inorganic fillers have been widely used by many research groups. Among them, the inclusion of inorganic fillers, such as nanoclay, carbon nanotube, zinc oxide, magnesium oxide, alumina, and titania (TiO_2_) into PLA matrix was considered as a useful and effective approach to enhance the properties of PLA [13,14].

By taking into account the unique properties of TiO_2_, the formation of nanocomposites composed of PLA and TiO_2_ nanoparticles would be a useful and effective approach to improve the properties of PLA. Up to date, this would be the first systematic review discussing in details the recent development in PLA/TiO_2_. Therefore, this article aims not only to identify the approaches used to enhance the dispersion of TiO_2_ in the PLA matrix but also to discuss the material properties of these composites.

## 2. Improvement of TiO_2_ Dispersion in PLA Matrix

**Figure 1:**
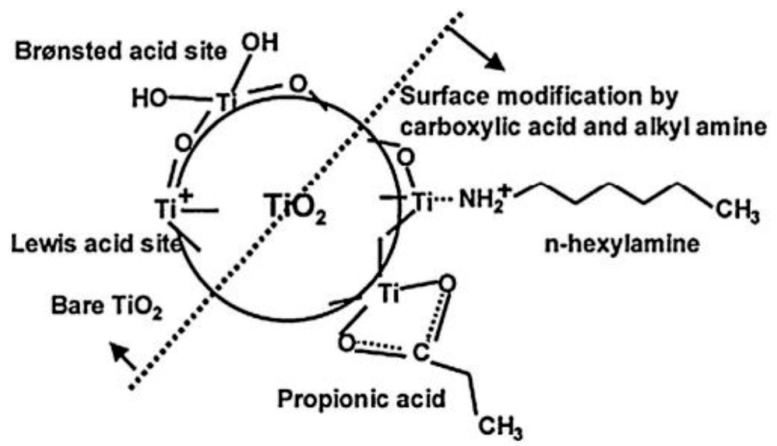
Surface functionalization of TiO_2_ nanoparticle by carboxylic acid and alkyl amine [17].

**Figure 2:**
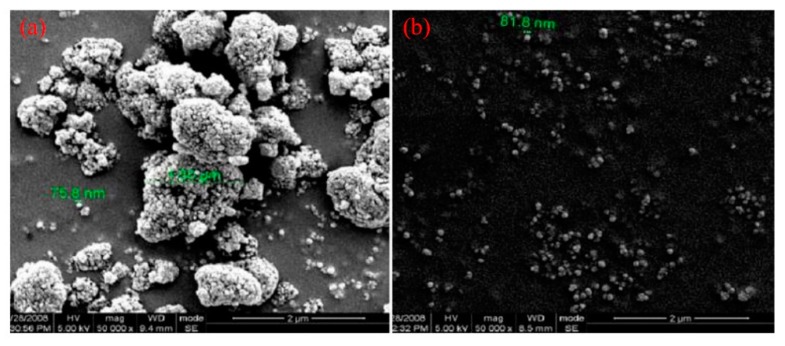
Scanning electron microscopy (SEM) images of TiO_2_ nanoparticles where (**a**) untreated TiO_2_ and (**b**) lactic acid-treated TiO_2_ (g-TiO_2_) [19].

Recently, much efforts have been devoted to improve dispersibility of nanoparticles within the polymer matrix by developing an adsorbed film on nanoparticles surface before fabricating polymer composites [15,16]. Accordingly, several methods, such as solution mixing, melt mixing, and in-situ polymerization have been utilized by several research groups [14]. As for PLA/TiO_2_ composites, it is mainly established that the mixing of untreated TiO_2_ nanoparticles with PLA causes their agglomeration within the PLA matrix [13]. Thus, the homogenous dispersion of TiO_2_ within PLA matrix would be needed in order to obtain high-performance composites. Here, the surface treatment or chemical functionalization of TiO_2_ nanoparticles are necessary in order to achieve better dispersibility. For instance, Nakayama and Hayashi [17] used propionic acid and long-chain alkyl amine in order to improve the dispersibility of TiO_2_ particles in PLA matrix (Figure 1). The carboxylic groups in PLA tended be bonded to TiO_2_ in a bridging bidentate mode [18,19,20]. In this direction, Luo et al. [19] chemically treated TiO_2_ nanoparticles (g-TiO_2_) in the existence of the lactic acid in order to enhance the dispersibility of nanoparticles in the PLA matrix. As compared to the untreated TiO_2_ nanoparticles, scanning electron microscopy (SEM) images in Figure 2 indicated that a better dispersibility in the PLA matrix could be achieved when g-TiO_2_ nanoparticles were added into PLA using melt mixing. As a result, the inclusion of g-TiO_2_ into PLA matrix not only increase PLA crystallinity but also improve the mechanical properties which is attributed to the good interfacial interactions between g-TiO_2_ and PLA matrix.

**Figure 3:**
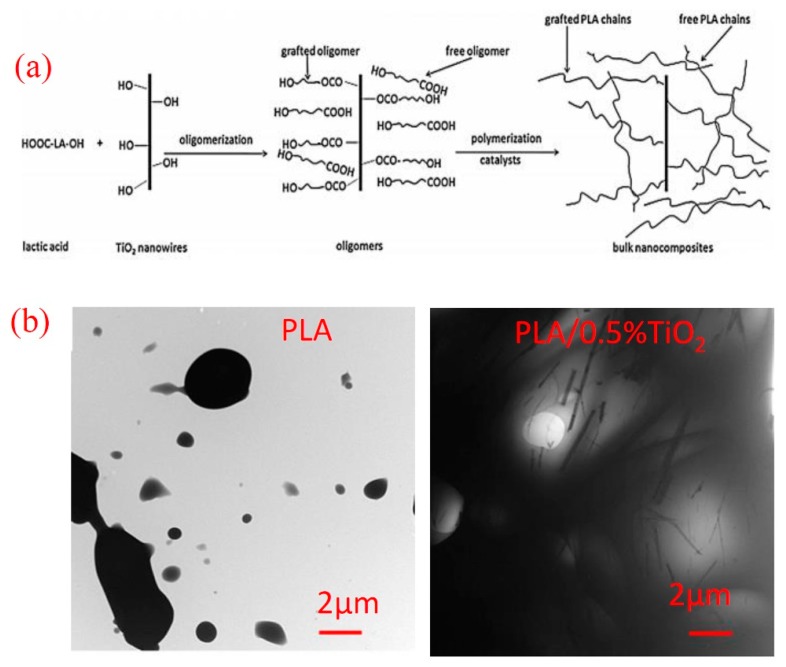
(**a**) Illustration showing the formation approach of polylactic acid (PLA)/TiO_2_ composites and (**b**) transmission electron microscopy (TEM) images of PLA and PLA/TiO_2_ composites containing 0.5 wt.% TiO_2_) [20].

As such, Li et al. [20] discovered that the chemical bonding between TiO_2_ nanowire surface and PLA chains by in situ melt polycondensation of LA could enhance the dispersibility of TiO_2_ in the PLA matrix. While the surface of TiO_2_ nanowires is chemically bonded with the carboxyl group of lactic acid, ester bonds could be formed because of the reaction between the hydroxyl groups in lactic acid with carboxyl group in another lactic acid. By removing the resulting water during the polycondensation process, a continuous growth of PLA chains on the surface of nanowire occurred, as demonstrated in Figure 3a. The dispersibility of the TiO_2_ nanowires in the PLA matrix was examined by transmission electron microscopy (TEM), as shown in Figure 3b. The TEM images presented in Figure 3b, implied that the distinct phase on TiO_2_ nanowire surfaces prevented the agglomeration of pure nanowires which led to a homogenous dispersion of nanowires in the PLA matrix. Accordingly, the thermal stability of PLA/TiO_2_ composites were better than that of the pure PLA.

As reported by Lu et al. [21], PLA chains can be grafted into TiO_2_ nanoparticles surface using in situ polymerization method. The lactic acid monomers were polymerized from the hydroxyl groups existing on the surface of TiO_2_ nanoparticles, in solution state using THF and chloroform as TiO_2_ and PLA solvents, respectively [20]. TEM results implied that the grafted TiO_2_ nanoparticles were distributed uniformly within the PLA matrix, leading to better chemical properties as compared to the case when un-grafted TiO_2_ nanoparticles were used.

Tabriz and Katbab [22] successfully modified the surface of TiO_2_ nanoparticles via melt mixing method in the presence of stannous chloride as catalyst. PLA chains were grafted onto nanoparticles surface through reactive melt mixing by an internal mixer using carboxylic acid terminal groups existing at the end of PLA chains. The composites films containing modified TiO_2_ exhibited higher antibacterial and higher amount of weight loss as compared to the films containing bare TiO_2_ nanoparticles.

## 3. Mechanical Properties

Several research groups have reported an enhancement in mechanical performance of PLA with the incorporation of TiO_2_ nanoparticles. Mechanical properties namely tensile strength (TS), Young’s modulus (YM), and elongation at break (EB) of PLA/TiO_2_ composites fabricated via melt mixing technique were examined by Alberton et al. [23]. While the values of TS and YM of the PLA were increased from ~53.66 MPa and ~3048 MPa into 58.28 MPa and 3237 MPa, respectively upon the incorporation of 1 wt.% TiO_2_ owing to the reinforcing effect of TiO_2_ nanoparticles, the EB value of PLA was found to be reduced from 3.56% to 3.00% with inclusion of 1 wt.% TiO_2_. According to Xiu et al. [24], the values of TS and EB for the PLA composite containing 10 wt.% TiO_2_ nanoparticles were slightly lower than those of neat PLLA. In addition, no obvious toughening effect on PLA was observed which was ascribed to the aggregation of TiO_2_ nanoparticles in PLA matrix. 

Athanasouliaa et al. [25] reported that incorporating 20 wt.% TiO_2_ into a PLA matrix could cause a large decrease in the values of TS and EB of the nanocomposites owing to poor dispersibility of TiO_2_ nanoparticles in the PLA matrix. However, the addition of 10 wt.% of TiO_2_ into PLA matrix caused minor changes in the values of YM of PLA. As demonstrated by Luo et al. [19], the surface functionalization of TiO_2_ by lactic acid prior to the melt mixing with PLA would be a useful strategy to improve the mechanical properties (EB and elasticity) of the resulting composites in comparison to the neat PLA. The impacts of TiO_2_ nanoparticles on the mechanical properties of PLA/sesbania composites were explored by Zhang and coworkers [26]. The optimal amount of TiO_2_ nanoparticles was 2 wt.%, at this content, the composites showed the maximum values of TS, bending strength, and EB. Foruzanmehr et al. [27] used TiO_2_-grafted flax fibers as a reinforcement agent for PLA. To achieve this purpose, a sol-gel coating technique was utilized to form a TiO_2_ film on the flax fiber. The modified fibers exhibited better adhesion and bonding toward PLA and thereby resulted in a three-fold improvement in the impact resistance of PLA. On the other hand, it was reported from the tensile test results that the oxidation of the flax fiber prior to the modification by TiO_2_ would induce the formation of a TiO_2_ inter-phase on the fiber. The inter-phase was not only led to reinforcement of the composites but also improved the interfacial connection between the fibers and the matrix.

**Figure 4:**
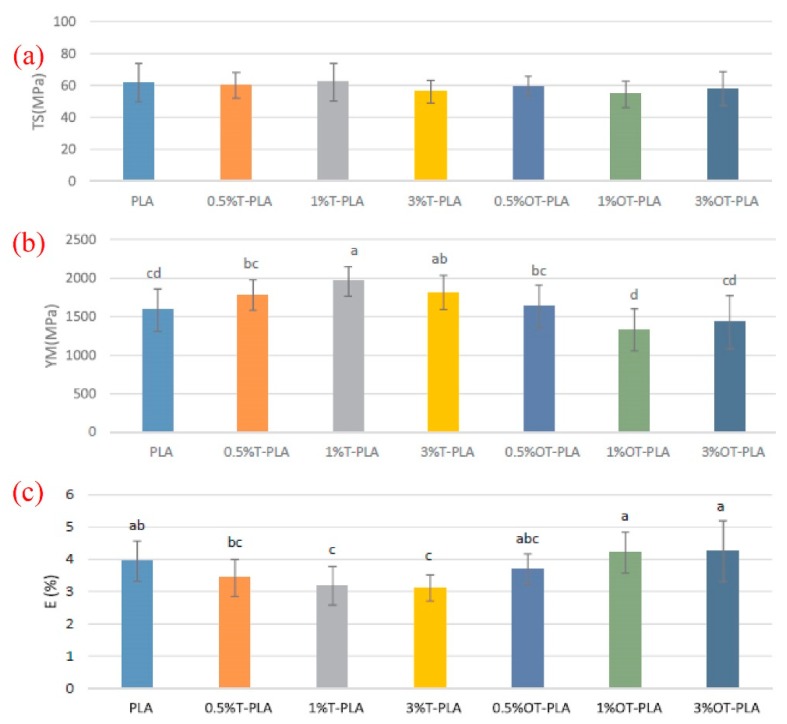
Mechanical results of PLA and PLA/TiO_2_ composites containing 0, 0.5, 1, 3 wt.% of either modified TiO_2_ or unmodified TiO_2_, where (**a**) TS, (**b**) YM and (**c**) EB (%) [28].

To increase the mechanical properties of PLA, Baek et al. [28] modified the surface of TiO_2_ by oleic acid. The mechanical results of the PLA composites comprising 0, 0.5, 1, and 3 wt.% of either modified TiO_2_ (named as OT-PLA) or unmodified TiO_2_ (named as T-PLA) were compared. As shown in Figure 4a, the value of the TS of the PLA was not greatly influenced by the inclusion of low contents of modified and unmodified TiO_2_. While the value of YM of PLA was increased by the incorporation of unmodified TiO_2_ as shown in Figure 4b for the T-PLA samples, the variations of YM with the addition of OT-TiO_2_ were insignificant. In addition, it was observed that the EB values of PLA with 1% OT-PLA and 3% OT-PLA were greatly higher than the counterparts with T-PLA (Figure 4c), implying that the mobility of PLA chains can be increased with the addition of OT-TiO_2_ into PLA, leading to higher values of EB.

In a work by Zhuang et al. [29], the mechanical properties of PLA/TiO_2_ nanocomposites were studied in terms of TS, EB, and YM. Before the preparation of PLA/TiO_2_ composites via in situ polymerization of LA, TiO_2_ was processed with γ-methacryloxypropyl trimethoxysilane as a coupling agent that increases the hydrophobicity of TiO_2_ nanoparticles, leading to a homogeneous dispersion within the PLA matrix. The mechanical results presented in Table 1 clearly indicated that the TS, EB, and YM of PLA were significantly improved when the amount of TiO_2_ was lower than 3 wt.% which was ascribed to the enhanced dispersibility of TiO_2_ nanoparticles in the PLA matrix. This finding suggested that the higher content of TiO_2_ nanoparticles (5 and 10 wt.%) in composites could lead to the acclamation of TiO_2_ nanoparticles in the PLA matrix. In the work of Marra et al. [30], PLA/TiO_2_ composite films were made by functionalizing TiO_2_ surfaces via fluorocarbons plasma treatment. The values of TS and EB were enhanced by 17% and 23%, respectively upon the inclusion of functionalized TiO_2_ nanoparticles. In contrast, the addition of 5 wt.% of the untreated TiO_2_ nanoparticles led to the deterioration of these properties by 12% and 15%, respectively

## 4. Thermal Properties

**Figure 5:**
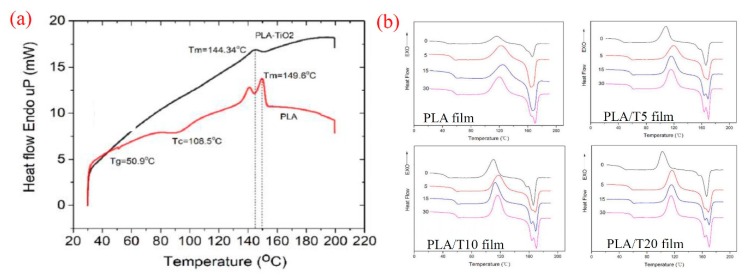
(**a**) Differential scanning calorimetry (DSC) curve of pure PLA and PLA/TiO_2_ composites [31] and (**b**) DSC curves of PLA, PL/T5, PLA/T10, and PLA/T20 composite films contacting with ethanol as food simulant at different period of time [32].

The thermal stability of the PLA composites acts as a crucial role in identification of the applications in which the composites can be used. Several investigations, therefore, have been performed on PLA’s composites with the purpose of controlling the thermal properties of these materials. The influence of TiO_2_ nanoparticles on the thermal properties of PLA/TiO_2_ composite films was studied by Mallick et al. [31]. As shown in Figure 5a, the melting temperature (T_m_) of PLA/TiO_2_ composite was less than that of neat PLA which was assigned to the role of TiO_2_ particles in disturbance the symmetry of the PLA chain structures and increasing the distance between the PLA chains. Therefore, the crystallization temperature (T_c_) and glass transition temperature (T_g_) observed in the differential scanning calorimetry (DSC) curves of neat PLA were disappeared in the curves of PLA/TiO_2_ composites. In the recent study by Yang and coworkers [32], 2 g of PLA was dissolved in 50 mL of dichloromethane under stirring condition. The PLA/TiO_2_ composite films were then fabricated by inserting various contents of TiO_2_ nanoparticles, such as 0, 5, 10, and 20 wt.% into the PLA solution under sonication for 30 min. Here, PLA, PLA/T5, PLA/T10, and PLA/T20 were donated to the films containing 0, 5, 10, and 20 wt.% of TiO_2_, respectively. The crystallinity percentage of the PLA phase in the PLA/TiO_2_ composites were found to be 14.2%, 15.8%, 18.2%, and 17.4% for PLA, PLA/T5, PLA/T10, PLA/T20 films, respectively. This result indicated that the crystallization degree of PLA can be improved with the incorporation of TiO_2_ nanoparticles. However, the crystallization degree tended to decrease when the amount of TiO_2_ nanoparticles exceeded 15 wt.% because of the agglomeration of TiO_2_ in the PLA matrix. On the other hand, the PLA, PLA/T5, and PLA/T10 films contacted with ethanol solution as alcoholic food for different periods of time, such as 0, 5, 15, and 30 days, exhibited a gradual increase in the values of T_g_ from day 0 to day 30 (Figure 5b). While, the T_g_ value of PLA/T20 film was increased in day 5 and then slightly decreased on day 15. The increase in the values of T_g_ implied that the amorphous phase was degraded in the early stages and the existence of more polymeric chains involved in the crystallization process. Based on the results obtained in this study, the authors suggested to use PLA/TiO_2_ composites as promising materials for food packaging applications.

The crystallinity of PLA and PLA/TiO_2_ composite films containing 1, 2, and 4 vol% of TiO_2_ was explored by Nomai et al. [33]. They found that the inclusion of TiO_2_ nanoparticles was useful to eliminate partially the reduction in the crystallinity of PLA after processing. Indeed, the presence of TiO_2_ nanoparticles nucleated PLA crystallization and cold crystallization, but decreased its spherulitic growth rate. This observation was checked by the three-fold value of the degree of crystallinity obtained by cold crystallization in the tested composites. The inclusion of 2 and 4 vol.% of TiO_2_ into PLA led to a slight decrease in the cold crystallization temperature (T_cc_) of the neat PLA from 130.2 °C to 129.5 and 128.2 °C, respectively, implying that TiO_2_ nanoparticles acted as nucleating agents or it could retard crystallization from the melt. Farhoodi et al. [34] examined the influence of TiO_2_ on crystallization behavior of PLA and reported that the degree of crystallinity of PLA/TiO_2_ composite can be enhanced and reached to the highest value at the low content of TiO_2_ (1–3 wt.%) because of the combined effects related to the nucleation and the growth restriction. 

Zhang et al. [35] used a vane extruder not only to promote the dispersibility of TiO_2_ nanoparticles in PLA matrix but also to reduce the degradability of the thermosensitive polymers. The addition of low content of TiO_2_, such as 0.5 or 1 wt.% into the PLA matrix increased the T_cc_ of the composites to a maximum value about 106 °C. This result suggested that the cold crystallization process can be inhibited by adding suitable amounts of TiO_2_. Based on the dynamic rheological and thermogravimetric results, it was confirmed that the stability of PLA can be enhanced with the inclusion of TiO_2_ nanoparticles. In another study on PLA and TiO_2_, it was reported that the inclusion of TiO_2_ nanoparticles into PLA would increase the crystallinity of the composites although the effects of such particles on the T_g_, T_cc_, and T_m_ were insignificant [36].

In the recent work by Athanasoulia and Tarantili [37], the crystallization kinetics of PLA/TiO_2_ composites fabricated via a twin-screw extruder were investigated isothermally at temperatures ranged from 100 to 120 °C. It was found that the crystallization rate at 100 and 110 °C was increased upon the inclusion of TiO_2_ into PLA matrix where the exothermic crystallization peaks tended to become narrower and crystallization occurred in shorter periods as compared to that in the neat PLA. At temperatures around 115 and 120 °C (closer to T_m_ of PLA), the crystallization process of PLA matrix would take place longer and the crystallization exothermic peaks tended to be broader in shape, resulting in longer periods to complete the crystallization. Thus, it was suggested that the crystallization mechanism of PLA was influenced not only by the inclusion and the amount of TiO_2_ nanoparticles, but also by the crystallization temperature chosen for testing. 

Buzarovska [38] mixed PLA with TiO_2_ nanoparticles functionalized with propanoic acid using solution casting technique. The effects of functionalization on the thermal properties of the PLA/functionalized TiO_2_ composites were examined and compared to those of PLA/untreated TiO_2_ composites. The degree of crystallinity in PLA composites containing functionalized TiO_2_ was significantly higher than that of PLA matrix. However, a discontinuous decrease of crystallinity was observed with an increment in the content of TiO_2_. In addition, *T_g_* of PLA was slightly increased with the inclusion of functionalized TiO_2_, while in composites containing untreated TiO_2_ the T_g_’s raise up to 5 °C in comparison to the neat PLA. As for PLA/TiO_2_ composite prepared by a melting process [39], T_c_ was raised from 106 °C for neat PLA to 120 °C for PLA/TiO_2_ composites, indicating that the inclusion of TiO_2_ nanoparticles triggers the crystallization process of PLA. However, at the higher contents of TiO_2_, the T_c_ could show a slight recovery as stated by Luo et al. [19] who reported that no noticeable change in the values of T_c_ can be noted when the 8 wt.% of TiO_2_ was inserted into the PLA matrix.

The catalytic effect of TiO_2_ and ZnO nanoparticles on the thermal stability of PLA was studied by Wang et al. [40] who demonstrated that the addition of TiO_2_ and ZnO into PLA matrix could reduce the activation energy for PLA required for pyrolysis and produced substantially higher degradation rate constant. Martín-Alfonso and coworkers [41] reported recently that the T_cc_, and the onset and maximum of thermal decomposition temperature of PLA tended to decrease with the addition of TiO_2_ and H_2_O_2_ into polymer matrix. This result was attributed to the formation of less stable compounds as a result of the photo-oxidation process.

## 5. Photocatalytic Properties

**Figure 6:**
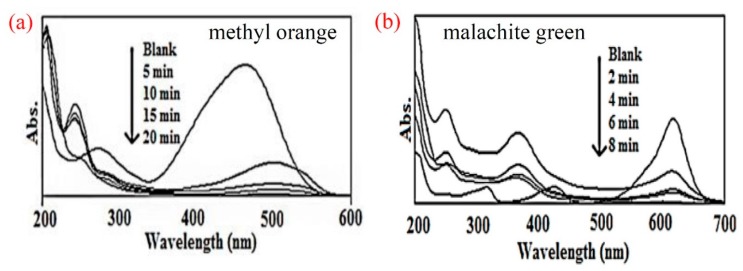
UV-Visible spectra of (**a**) methyl orange and (**b**) malachite green after exposure to UV irradiation in different periods of time [43].

Owing to its photocatalytic activity, TiO_2_ nanoparticle with high specific surface area can degrade various organic compounds, making it a suitable material for many photocatalytic applications [42]. To examine the photocatalytic performance of PLA/TiO_2_ composites, Shaikh and coworkers used methyl orange and malachite green as anionic and cationic dyes, respectively [43]. The results revealed that the two dyes tended to adsorb on the surface of the catalyst which resulted in a decrease in the concentration of catalyst sonicated with dye in dark by 9.2% and 21.5% for methyl orange and malachite green, respectively. However, the exposure to UV light would make both the dyes colorless. From UV visible spectra shown in Figure 6a,b, it was found that a complete discoloration of a 10^−4^ M solution of methyl orange was noted in 20 min whereas that of malachite green was noticed in 8 min with 50 mg of the PLA/TiO_2_ photocatalyst. The authors suggested that the photodegradation mechanism can be summarized by the Equations (1)–(5) considering the fact that the addition of KI could significantly inhibit the degradation of dyes.

(1) $$Dye+h\upsilon \to Dye^{*}$$

(2) $$Dye^{*}+TiO_{2}\to Dye^{\cdot +}+TiO_{2}\left(e^{-}\right)$$

(3) $$TiO_{2}\left(e^{-}\right)+{\left(O_{2}\right)}_{ads}=TiO_{2}+O_{2}^{\cdot -}$$

(4) $$O_{2}^{\cdot -}+Dye^{\cdot +}\to Degradation\;products$$

(5) $$I^{-}+Dye^{\cdot +}\to Dye+I\to solar\;cells$$

Zhu et al. [44,45] fabricated active films incorporating TiO_2_ nanoparticles into PLA films via compression and extrusion methods. Among all films subjected to UV irradiation for 10 h, the PLA film containing 10 wt.% of TiO_2_ particles exhibited a decolorization degree of 80%, suggesting a good improvement in photocatalytic activity can be obtained via the incorporation of TiO_2_ into PLA film. 

Hou et al. [46] successfully prepared TiO_2_-loaded PLA composite fibers through the ultrasonic irradiation induced in situ deposition of TiO_2_ nanoparticles. Considering the fact that TiO_2_ nanoparticles were well distributed in the surface of PLA fibers, the specific surface area of PLA was enlarged from 12.9 m^2^/g to 64.8 m^2^/g when TiO_2_ nanoparticles attached to the surface of PLA. The photocatalytic activity of the fibers obtained from pure PLA or PLA/TiO_2_ composite was confirmed by the degradation of methyl orange up to 5% and 76%, respectively under UV irradiation for 12 h.

## 6. Antimicrobial Properties

In view of the non-ionization nature of TiO_2_ nanoparticles, the inclusion of TiO_2_ into the PLA matrix was reported to be efficient against miscellaneous bacterial strains and suggested to be used instead of Ag nanoparticles [47]. Generally, the migration phenomenon of nanoparticles is a critical factor to evaluate the safety and relevance of the PLA/TiO_2_ composites [48]. The antimicrobial activity of PLA composite films can be conducted by direct contact rather than sustained release of active materials to fresh products [49]. For instance, Li et al. [50] demonstrated that the amounts of TiO_2_ and Ag nanoparticles migrated from PLA/TiO_2_ and PLA/TiO_2_ + Ag composite films to cheese specimens were too much lower than the migration limit proposed by European Food Safety Agency for food contact materials. Thus, the PLA/TiO_2_ composites could be utilized safely as antimicrobial food packaging films.

The impacts of TiO_2_ nanoparticles on the antimicrobial properties of PLA/TiO_2_ were reported by Li et al. [51]. The PLA composites containing 1 or 5 wt.% TiO_2_ nanoparticles were fabricated via a solvent mixing method. Two types of bacterial, such as Escherichia coli (E. *coil*) and *Listeria* were selected in order to discover the antimicrobial activity of the composites. The results of antimicrobial tests implied that the growth of the two types of bacteria was not affected by the film fabricated only from pure PLA. After 1 day, the amounts of the two tested bacteria were increased to 8.94 and 9.12 log10CFU/mL for E. *coli* and *Listeria* monocytogenes, respectively. In contrast, the value of *E. coli* was reduced to 4.35 and 3.45 with addition of 1 and 5 wt.% of TiO_2_ into PLA matrix, respectively. Whereas, the value of *Listeria* bacteria was reduced to 4.15 and 3.67 upon the addition of 1 and 5 wt.% of TiO_2_ into PLA matrix, respectively. This finding suggested that the inclusion of TiO_2_ into PLA matrix can effectively inhibit bacterial reproduction. These findings were in accordance with those obtained by Falco et al. [52] where excellent antibacterial properties against the evolution of microbial biofilms were obtained upon the application of TiO_2_ coatings on aluminum substrates. Based on earlier investigation by Lian and coworkers [53], microbes could be killed by the low size of TiO_2_ nanoparticles which also produced many electron-hole pairs, triggering redox reactions on those microorganisms. Díez-Pascual [54] postulated that a 3.0 wt.% would be the lowest amount of TiO_2_ needed for efficient microbial growth inhibition. 

Fonseca et al. [39] assessed the antimicrobial and antifungal characteristics of PLA/TiO_2_ composites against E. *coli* and A. *fumigatus* without and with UV irradiation. The PLA composites containing 8 wt.% TiO_2_ were found to be effective against E. *coli* and A. *fumigatus* with 82.4% and 52.6% reduction, respectively, irrespective of UV irradiation. However, the PLA/TiO_2_ composite under irradiation condition exhibited a reduction of E. *coli* and A. *fumigatus* of 94.3% and 99.9%, respectively, indicating that PLA/TiO_2_ composites have the ability to be employed as promising materials in food packaging or medical applications. Very recently, Feng and coworkers [55] reported that incorporation of 0.75 wt.% TiO_2_ into PLA matrix can lead to significant improvement in the antibacterial performance of PLA where inhibition areas of (~4.86 and ~3.69 mm) and (~4.63 and ~5.98 mm) were obtained for *E. coli* and *S. aureus*, respectively.

**Figure 7:**
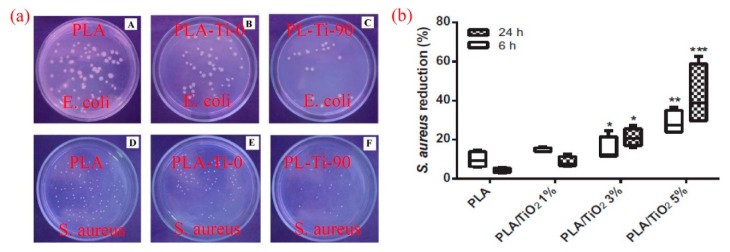
(**a**) Antimicrobial activity of PLA PLA/TiO_2_ composites against E. *coli* and S. *aureus* [56], (**b**) S. *aureus* reduction as a function of TiO_2_ content [57].

According to Gupta et al. [56], PLA/TiO_2_ nanofibers were made using a hydrothermal process that not only produces the anatase phase but also helps to decorate the fiber surface. As a result, the fibers possessed antimicrobial activity against E. *coli* and S. *aureus* at the high TiO_2_ content, which affected biocidal activity during the following hours (Figure 7a). Further studies by Toniatto and coworkers [57] disclosed that PLA/TiO_2_ fibers maintained their antibacterial efficiency against S. *aureus* at low contents of TiO_2_ (1–5 wt.%) without proof of in vitro cytotoxicity (24–168 h, fibroblast cell line) (Figure 7b). As such, Dural-Erem [58] incorporated TiO_2_ nanoparticles in the form anatase (0.1 to 5 wt.%) into PLA matrix via melt mixing process. The composites films exhibited good bacteriostatic performance against Klebsiella *pneumoniae* (ATCC 4352) and Staphylococcus *aureus* (ATCC 6538). The authors attributed this result to the fact that the adsorption of water molecules on the composites surface would induce the release of active oxygen species from TiO_2_ nanoparticles.

## 7. Degradation Behavior

**Figure 8:**
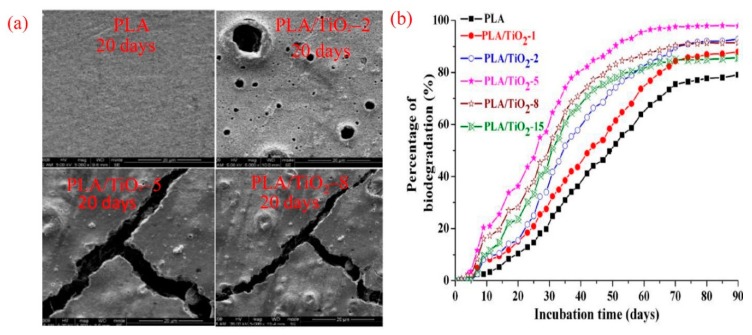
(**a**) SEM images of PLA and PLA/TiO_2_-2, PLA/TiO_2_-5, and PLA/TiO_2_-8 composites after 20 days incubation time and (**b**) the percentage of biodegradation with respect to incubation time for pure PLA and PLA/TiO_2_ composites [59].

Controlling the degradation behavior of PLA composites is a key consideration from the scientific and industrial perspectives. In general, the incorporation of TiO_2_ nanoparticles was found to be an effective approach to monitor the degradation behavior of PLA in different media. The degradation of PLA/TiO_2_ composites can be classified into several types, such as biodegradation, thermal degradation, photodegradation under UV irradiation, hydrolytic degradation, and enzymatic degradation. For example, Luo and coworkers [59] studied the biodegradability of PLA/TiO_2_ composites formed by the melt mixing of PLA with functionalized g-TiO_2_ via a twin-screw extruder. The content of TiO_2_ in the composites was 0.5, 1.0, 2.0, 5.0, 8.0, and 15.0 wt.%. The prepared composites were subjected to biodegradation tests under controlled compositing conditions for three months. SEM images presented in Figure 8a for neat PLA and PLA/TiO_2_ composites after incubation periods for 20 days indicated that a considerable degradation of PLA/TiO_2_ composite can occur in comparison to that of PLA. This was characterized by the presence of deep cracks and large voids on the surface of PLA/TiO_2_ composites as a result of the hydrolysis of PLA and microorganisms activities, indicating chain loss and surface erosion of the composites. In addition, it was found that the amounts of TiO_2_ would accelerate the initial phase of degradation and enhanced the amount of CO_2_ generated at the end of incubation periods. After 80 days of incubation, the biodegradation percentage of PLA was found to be 78.9% which was lower than that of PLA/TiO_2_ composites which were 86.9, 92.0, 97.8, 91.3, and 85% for the composites containing 1, 2, 5, 8, and 15 wt.% TiO_2_, respectively (Figure 8b). 

As to the hydrolytic degradation of PLA, it was reported that the hydrolysis of PLA in the presence of nanofillers can be affected by several factors related to the morphology, dispersion, and hydrophilicity of nanofillers [60]. Therefore, the hydrolytic degradation of PLA can be delayed or favored based on the type of nanofillers [61]. Previous studies indicated that the degradation efficiency of a PLA was improved by the incorporation of TiO_2_ nanoparticles. The long-term hydrolytic degradation of PLA/TiO_2_ composites (1–15 wt.% TiO_2_) in a phosphate buffer solution of pH 7.4 at 37 °C was examined by Luo et al. [15]. By inclusion of TiO_2_ nanoparticles into the PLA matrix, a significant change in the morphology of composites was observed, indicating that the bulk erosion process was altered through the initial inhomogeneous degradation at the PLA matrix-TiO_2_ interface. The inhomogeneous degradation and bulk erosion process of PLA were sped up with increasing the amount and dispersibility of TiO_2_ nanoparticles during the degradation. For example, the hydrolysis of neat PLA was accelerated by the addition of 8 and 15 wt.% of TiO_2_ matrix since the weight losses for PLA were increased from values lower than 2% to values of 8 to 15 wt.% upon the inclusion of 8 and 15 wt.% of TiO_2_ into the polymer matrix, respectively. This result was connected to the hydrophilicity of TiO_2_ as well as the high-water absorption of composites.

The photodegradation of PLA/TiO_2_ composites, which occurs under UV light exposure, was suggested to be the primary causes of damage of PLA in ambient environments. According to earlier investigation by Luo et al. [62], the anatase nanoparticles were grafted by lactic acid oligomer via solution condensation reaction. The grafted anatase (termed as g-TiO_2_) (0 to 15 wt.%) were then melt mixed with PLA using a Brabender for 3 min at 185 °C. The photodegradation of PLA and PLA/g-TiO_2_ was studied under UV irradiation at room temperature without humidity rate control. It was confirmed that the photodegradability of PLA can be controlled by adjusting the amounts of g-TiO_2_ distributed in PLA matrix. For example, the photodegradability of PLA was increased remarkably upon incorporation of 0.5 wt.% g-TiO_2_ nanoparticles. While the lower contents of TiO_2_ (≤2 wt.%) led to the increase in the weight losses of the composites; opposite behavior was found when the higher contents of TiO_2_ were added into PLA matrix. Since the PLA/TiO_2_-2 composite showed the fastest weight loss rate, the photocatalytic degradation efficiency of this composite was superior to other composites.

Marra et al. [63] studied the photodegradation of PLA and PLA/TiO_2_ composites exposed to UV-accelerated weathering tester with an average irradiance of 20 W·m^−2^. The temperature and humidity were controlled to be 40 °C and 25%, respectively. It was demonstrated that the weight loss in PLA/TiO_2_ composites was significantly slower than that in the neat PLA. The UV degradation of PLA/TiO_2_ composites can be reached 50% after 40 days of UV exposure while the neat PLA was completely degraded after only 17 days of UV exposure, indicating that UV degradation of PLA can be decreased significantly by the inclusion of TiO_2_ particles. In addition, the authors proved that the amount of TiO_2_ in the composites could control the hydrolytic degradation of PLA in 1 M NaOH where the PLA/TiO_2_ composites showed higher weight lost with respect of time than neat PLA. Similar results were obtained by Buzarovska and Grozdanov [36].

Nakayama and Hayashi [17] fabricated PLA/TiO_2_ composite films by adding modified TiO_2_ nanoparticles into the PLA matrix. The degradation of composite films by UV irradiation was easier than that in the neat PLA films. Zhuang et al. utilized in situ polymerization approach to fabricate PLA/TiO_2_ composites with different content of TiO_2_ [29]. The PLA/TiO_2_ composites showed higher photodegradability when subjected to UV irradiation test. In contrast to results obtained by Nakayama and Hayashi [17], Buzarovska [38] found that the functionalization of TiO_2_ particles by propanoic acid had insignificant effects on the photodegradability of PLA/TiO_2_ composites prepared by solution mixing method since the modified TiO_2_ nanoparticles were not well distributed within the PLA matrix. On the other hand, Man et al. [64] found that the photodegradation of PLA composites including TiO_2_ in the form of anatase can be influenced by the thickness of the films. From UV absorbance results, the thick films exhibited a UV shielding influence while the degradation was accelerated in the case of the films with low thickness. 

The enzymatic degradation of PLA/TiO_2_ composites has extensively been studied because of the fact that this type of degradation usually does not require high temperatures to be accomplished. For example, Buzarovska and Grozdanov [36] examined the enzymatic degradation of PLA/TiO_2_ composites in α-amylase solutions at 37 °C. The extent of enzymatic degradation of the composites containing 0.5 wt.% TiO_2_ after 126 h of exposure was found to be higher than other composites, indicating that a diffusion-controlled process was the main factor affecting the degradation process of PLA because of the fact that higher content of TiO_2_ could suppress the diffusion process by blocking the diffusion of α-amylase molecules.

## 8. Potential Applications of PLA/TiO_2_ Composites

PLA/TiO_2_ composites can be used in many biomedical and industrial fields because of their excellent properties as we discussed above. For example, PLA/TiO_2_ composites are promising materials for food packaging applications [34,35,39,50,65,66]. Based on the experimental results of Chi and coworkers [65], PLA/TiO_2_ composites were suggested to be promising materials for food preservation in order to improve the shelf life of fruits and vegetables. The functionalization of TiO_2_ nanoparticles, e.g., with oleic acid can help to obtain promising scaffolds for drug delivery applications [67,68]. Song et al. [69,70] fabricated PLA nanofibers via electrospinning method and then combined with TiO_2_ nanoparticles by adding them into the working medium where a glassy carbon electrode was utilized as the working electrode. The fabricated PLA/TiO_2_ composites can effectively promote the relative biorecognition of daunorubicin to DNA.

Owing to the outstanding antimicrobial activity of porous honeycomb fabricated via breath-figure method, PLA/rutile composite was suggested as an effective wound healing dressing material [71]. In addition, high-performance membrane devices could be designed through controlling the morphologies of PLA/TiO_2_ composites [72]. Based on the air filtration results obtained by Wang and coworkers on PLA/TiO_2_ composites, it would be possible to make a fibrous filter with a high filtration efficiency and energy-saving ability [73]. The deposition of TiO_2_ on the surface of carbon nanotube before mixing with PLA would also lead to fabricate disposable electronics [74]. Finally, PLA/TiO_2_ composites can be used as promising materials in catalyst applications due to their excellent catalytic properties [75].

## 9. Conclusions

This article mainly introduced the research status of TiO_2_ nanoparticles to improve the material properties of biodegradable PLA. In general, the material properties of PLA/TiO_2_ composites could be influenced by several factors connected to the processing method, distribution of TiO_2_ particles, size and content of TiO_2_ particles. The homogeneous distribution of TiO_2_ in the PLA matrix would be challenging because of the fact that TiO_2_ particles tended to be agglomerated in the PLA matrix in particular when the content of TiO_2_ is higher than 3 wt.%. Thus, the functionalization of TiO_2_ particles prior to mixing with PLA would be necessary in order to solve this problem. In general, the mechanical properties namely Young’s modulus and tensile strength of PLA/TiO_2_ composites could be enhanced because of the reinforcement effect of TiO_2_ in PLA matrix. In addition, the toughness of PLA could be increased upon the addition of functionalized TiO_2_ particles into the polymer matrix. The incorporation of TiO_2_ particles acting as nucleating agents could also improve the thermal stability of PLA. The relatively low degradation efficiency of a PLA matrix can be remarkably improved by the incorporation of TiO_2_ nanoparticles. The PLA/TiO_2_ composites can be utilized as antibacterial materials. Finally, we hope that this review article can help readers with a wide range of backgrounds to comprehend the impacts of TiO_2_ nanoparticles on the performance and applications of PLA composites.

## Figures and Tables

**Table 1:** Mechanical properties of PLA and PLA/TiO_2_ composites [29].

	PLA	PLA/TiO_2_-1	PLA/TiO_2_-3	PLA/TiO_2_-5	PLA/TiO_2_-10
TiO_2_ content (%)	0	1	3	5	10
TS (MPa)	9.37	9.45	17.2	10.5	3.35
EB (%)	245.3	250.0	261.8	178.6	39.4
YM(MPa)	12.3	138.3	287.5	253.5	202.0

## References

[1] Chen X., Mao S.S. (2007). Titanium dioxide nanomaterials: Synthesis, properties, modifications, and applications. Chem. Rev..

[2] Docekal B., Vojtková B. (2007). Determination of trace impurities in titanium dioxide by direct solid sampling electrothermal atomic absorption spectrometry. Spectrochim. Acta Part B At. Spectrosc..

[3] Allodi V., Brutti S., Giarola M., Sgambetterra M., Navarra M.A., Panero S., Mariotto G. (2016). Structural and spectroscopic characterization of a nanosized sulfated TiO_2_ filler and of nanocomposite nafion membranes. Polymers.

[4] Zapata P.A., Palza H., Rabagliati F.M. (2012). Novel antimicrobial polyethylene composites prepared by metallocenic “in-situ” polymerization with TiO_2_ based nanoparticles. J. Polym. Sci. A Polym. Chem..

[5] Li Y., Chen C., Li J., Sun X. (2014). Photoactivity of poly(lactic acid) nanocomposites modulated by TiO_2_ nanofillers. J. Appl. Polym. Sci..

[6] Wang Z., Wang X., Xie G., Li G., Zhnag Z. (2006). Preparation and characterization of polyethylene/TiO_2_ nanocomposites. Compos. Interfaces.

[7] Althan M., Yildirim H. (2012). Mechanical and Antibacterial Properties of Injection Molded Polypropylene/TiO_2_ Nano-Composites: Effects of Surface Modification. J. Mater. Sci. Technol..

[8] Xuefeng L., Shijie D., Han Y. (2012). Fabrication and properties of PVA-TiO_2_ hydrogel composites. Procedia Eng..

[9] Hanemann T., Szabo D.V. (2010). Polymer-nanoparticle composites: From synthesis to modern applications. Materials.

[10] Basu A., Nazarkovsky M., Ghadi R., Khan W., Domb A.J. (2017). Poly(lactic acid)-based nanocomposites. Polym. Adv. Technol..

[11] Xu N., Shi Z., Fan Y., Dong J., Sji J., Hu M.Z.C. (1999). Effects of particle size of TiO_2_ on photocatalytic degradation of methylene blue in aqueous suspensions. Ind. Eng. Chem. Res..

[12] Hamad K., Kaseem M., Ayyoob M., Joo J., Deri F. (2018). Polylactic acid blends: The future of green, light and tough. Prog. Polym. Sci..

[13] Raquez J.M., Habibi Y., Murariu M., Doubois P. (2013). Polylactide (PLA)-based nanocomposites. Prog. Polym. Sci..

[14] Kaseem M., Hamad K., Deri F., Ko Y.G. (2017). A review on recent researches on polylactic acid/carbon nanotube composites. Polym. Bull..

[15] Luo Y.B., Wang X.L., Wang Y.Z. (2012). Effect of TiO_2_ nanoparticles on the long-term hydrolytic degradation behavior of PLA. Polym. Degrad. Stab..

[16] Kaseem M., Hamad K., Ko Y.G. (2016). Fabrication and materials properties of polystyrene/carbon nanotube (PS/CNT) composites: A review. Eur. Polym. J..

[17] Nakayama N., Hayashi T. (2007). Preparation and characterization of poly(l-lactic acid)/ TiO_2_ nanoparticle nanocomposite films with high transparency and efficient photodegradability. Polym. Degrad. Stabil..

[18] Hojjati B., Sui R., Charpentier P.A. (2007). Synthesis of TiO_2_/PAA nanocomposite by RAFT polymerization. Polymer.

[19] Luo Y.B., Li W.D., Wang X.L., Xu D.Y., Wang Y.Z. (2009). Preparation and properties of nanocomposites based on poly (lactic acid) and functionalized Tioacta. Acta Mater..

[20] Li Y., Chen C., Li J., Sun X.S. (2011). Synthesis and characterization of bio-nanocomposites of poly(lactic acid) and TiO_2_ nanowires by in situ polymerization. Polymer.

[21] Lu X. (2008). Nanocomposites of poly(L-lactide) and surface-grafted TiO_2_ nanoparticles: Synthesis and characterization, People’s Republic of China. Eur. Polym. J..

[22] Tabriz K.R., Katbab A.A. (2017). Preparation of modified-TiO_2_/PLA nanocomposite films: Micromorphology, photo-degradability and antibacterial studies. AIP Conf. Proc..

[23] Alberton J., Martelli S.M., Fakhouri F.M., Soldi V. (2014). Mechanical and moisture barrier properties of titanium dioxide nanoparticles and halloysite nanotubes reinforced polylactic acid (PLA). IOP Conf. Ser. Mater. Sci. Eng..

[24] Xiu H., Bai H.W., Huang C.M., Xu C.L., Li X.Y., Fu Q. (2013). Selective localization of titanium dioxide nanoparticles at the interface and its effect on the impact toughness of poly(L-lactide)/poly(ether)urethane blends. Express. Polym. Lett..

[25] Athanasoulia I.G., Mikropoulou M., Karapati S., Tarantili P., Trapalis C. (2018). Study of thermomechanical and antibacterial properties of TiO_2_/poly(lactic acid) nanocomposites. Mater. Today Proc..

[26] Zhang Q., Li D., Zhang H., Su G., Li G. (2018). Preparation and properties of poly(lactic acid)/sesbania gum/nano-TiO_2_ composites. Polym. Bull..

[27] Foruzanmehr M., Vuillaume P.Y., Elkoun S., Robert M. (2016). Physical and mechanical properties of PLA composites reinforced by TiO_2_ grafted flax fibers. Mater. Des..

[28] Baek N., Kim Y.T., Marcy J.E., Duncan S.E., O’Keefe S.F. (2018). Physical properties of nanocomposite polylactic acid films prepared with oleic acid modified titanium dioxide. Food. Packag. Shelf..

[29] Zhuang W., Liu J., Zhang J.H., Hu B.X., Shen J. (2009). Preparation characterization and properties of TiO_2_/PLA nanocomposites by in situ polymerization. Polym. Compos..

[30] Marra A., Silvestre C., Kujundziski A.P., Chamovska D., Duraccio D. (2017). Preparation and characterization of nanocomposites based on PLA and TiO_2_ nanoparticles functionalized with fluorocarbons. Polym. Bull..

[31] Mallick S., Ahmad Z., Touati F., Bhadra J., Shakoor R.A., Al-Thani N.J. (2018). PLA-TiO_2_ nanocomposites: Thermal, morphological, structural, and humidity sensing properties. Ceram. Int..

[32] Yang C., Zhu B., Wang J., Qin Y. (2019). Structural changes and nano-TiO_2_ migration of poly(lactic acid)-based food packaging film contacting with ethanol as food simulant. Int. J. Biol. Macromol..

[33] Nomai J., Suksut B., Schlab A.K. (2015). Crystallization behavior of poly(lactic acid)/titanium dioxide nanocomposites. Int. J. Appl. Sci. Technol..

[34] Farhoodi M., Daddashi S., Mohammad M.A., Mousavi A., Djomeh Z. (2012). Influence of TiO_2_ Nanoparticle Filler on the Properties of PET and PLA Nano composites. Polymer (Korean) ISSN.

[35] Zhang H., Huang J., Yang L., Chen R., Zou W., Lin X., Qu J. (2015). Preparation, characterization and properties of PLA/TiO_2_ nanocomposites based on a novel vane extruder. RSC Adv..

[36] Buzarovska A., Grozdanov A. (2011). Biodegradable poly(l-lactic acid)/TiO_2_ nanocomposites: Thermal properties and degradation. J. Appl. Polym. Sci..

[37] Athanasoulia I.G.I., Tarantili P.A. (2019). Thermal transitions and stability of melt mixed TiO_2_/poly(L-lactic acid) nanocomposites. Polym. Eng. Sci..

[38] Buzarovska A. (2013). PLA Nanocomposites with Functionalized TiO_2_ Nanoparticles. Polym. Plast. Technol. Eng..

[39] Fonseca C., Ochoa A., Ulloa M.T., Alvarez E., Canales D., Zapata P.A. (2015). Poly(lactic acid)/TiO_2_ nanocomposites as alternative biocidal and antifungal materials. Mater. Sci. Eng. C.

[40] Wang X.J., Huang Z., Wei M.Y., Lu T., Nong D.D., Zhao J.X., Gao X.Y., Teng L.J. (2019). Catalytic effect of nanosized ZnO and TiO_2_ on thermal degradation of poly (lactic acid) and isoconversional kinetic analysis. Thermochim. Acta..

[41] Martín-Alfonsoa J.E., Urbanob J., Cuadria A.A., Franco J.M. (2019). The combined effect of H_2_O_2_ and light emitting diodes (LED) process assisted by TiO_2_ on the photooxidation behavior of PLA. Polym. Test..

[42] Joost U., Juganson K., Visnapuu M., Mortimer M., Kahru A., Nõmmiste E., Joost U., Kisand V., Ivask A. (2015). Photocatalytic antibacterial activity of nano-TiO_2_ (anatase)-based thin films: Effects on Escherichia coli cells and fatty acids. J. Photochem. Photobiol. B Biol..

[43] Shaikh T., Rathore A., Kaur H. (2017). Poly (lactic acid) grafting of TiO_2_ nanoparticles: A shift in dye degradation performance of TiO_2_ from UV to solar light. Chem. Select.

[44] Zhu Y., Buonocore G.G., Lavorgna M., Ambrosio L. (2011). Poly(lactic acid)/titanium dioxide nanocomposite films: Influence of processing procedure on dispersion of titanium dioxide and photocatalytic activity. Polym. Compos..

[45] Zhu Y., Buonocore G.G., Lavorgna M. (2012). Photocatalytic activity of PLA/TiO_2_ nanocomposites and TiO_2_-active multilayered hybrid coatings. Ital. J. Food. Sci..

[46] Hou X.B., Cai Y.B., Mushtaq M., Song X., Yang Q., Huang F., Wei Q. (2018). Deposition of TiO_2_ nanoparticles on porous polylactic acid fibrous substrates and its photocatalytic capability. J. Nanosci. Nanotechnol..

[47] Garcia C.V., Shin G.H., Kim J.T. (2018). Metal oxide-based nanocomposites in food packaging: Applications, migration, and regulations. Trends. Food. Sci. Technol..

[48] Girdthep S., Worajittiphon P., Molloy R., Lumyong S., Leejarkpai T., Punyodom W. (2014). Biodegradable nanocomposite blown films based on poly(lactic acid) containing silver-loaded kaolinite: A route to controlling moisture barrier property and silver ion release with a prediction of extended shelf life of dried longan. Polymer..

[49] Lantano C., Alfieri I., Cavazza A., Corradini C., Lorenzi A., Zucchetto N., Montenero A. (2014). Natamycin based sol-gel antimicrobial coatings on polylactic acid films for food packaging. Food. Chem..

[50] Li W., Li L., Zhang H., Yuan M., Qin Y. (2018). Evaluation of PLA nanocomposite films on physicochemical and microbiological properties of refrigerated cottage cheese. J. Food. Process. Pres..

[51] Li W., Zhang C., Chi H., Li L., Lan T., Han P., Chen H., Qin Y. (2017). Development of antimicrobial packaging film made from poly(lactic acid) incorporating titanium dioxide and silver nanoparticles. Molecules.

[52] De Falco G., Porta A., Petrone A.M., Del Gaudio P., El Hassanin A., Commodo M., Minutolo P., Squillace A., D’Anna A. (2017). Antimicrobial activity of flame-synthesized nano-TiO_2_ coatings. Environ. Sci. Nano.

[53] Lian Z., Zhang Y., Zhao Y. (2016). Nano-TiO_2_ particles and high hydrostatic pressure treatment for improving functionality of polyvinyl alcohol and chitosan composite films and nano-TiO_2_ migration from film matrix in food simulants. Innov. Food Sci. Emerg. Technol..

[54] Pascual A.M.D., Diez-Vicente A.L. (2014). Effect of TiO_2_ nanoparticles on the performance of polyphenylsulfone biomaterial for orthopedic implants. J. Mater. Chem. B.

[55] Feng S., Zhang F., Ahmed S., Liu Y. (2019). Physico-mechanical and antibacterial properties of PLA/TiO_2_ composite materials synthesized via electrospinning and solution casting processes. Coatings.

[56] Gupta K.K., Mishra P.K., Srivastava P., Gangwar M., Nath G., Maiti P. (2013). Hydrothermal in situ preparation of TiO_2_ particles onto poly(lactic acid) electrospun nanofibers. Appl. Surf. Sci..

[57] Toniatto T.V., Rodrigues B.V.M., Marsi T.C.O., Ricci R., Marciano F.R., Webster T.J., Lobo A.O. (2017). Nanostructured poly (lactic acid) electrospun fiber with high loadings of TiO_2_ nanoparticles: Insights into bactericidal activity and cell viability. Mater. Sci. Eng. C.

[58] Dural-Erem A., Erem H.H., Ozcan G., Skrifvars M. (2015). Anatase titanium dioxide loaded polylactide membranous films: Preparation, characterization, and antibacterial activity assessment. J. Text. I..

[59] Luo Y., Lin Z., Guo G. (2019). Biodegradation assessment of poly (lactic acid) filled with functionalized Titania nanoparticles (PLA/TiO_2_) under compost conditions. Nanoscale Res. Lett..

[60] Williams D.F. (1981). Enzymatic hydrolysis of polylactic acid. Eng. Med..

[61] Oda Y., Yonetsu A., Urakami T., Tonomura K. (2000). Degradation of polylactide by commercial proteases. J. Polym. Environ..

[62] Luo Y.B., Cao Y.Z., Guo G. (2018). Effects of TiO_2_ nanoparticles on the photodegradation of poly (lactic acid). J. Appl. Polym. Sci..

[63] Marra A., Cimmino S., Silvestre C. (2017). Effect of TiO_2_ and ZnO on PLA degradation in various media. Adv. Mater. Sci..

[64] Man C., Zhang C., Liu Y., Wang W., Ren W., Jiang L., Reisdorffer F., Nguyen T.P., Dan Y. (2012). Poly (lactic acid)/titanium dioxide composites: Preparation and performance under ultraviolet irradiation. Polym. Degrad. Stab..

[65] Chi H., Song S., Luo M., Zhang G., Li W., Li L., Qin Y. (2019). Effect of PLA nanocomposite films containing bergamot essential oil, TiO_2_ nanoparticles, and Ag nanoparticles on shelf life of mangoes. Sci. Hortic..

[66] Segura Gonzalez E.A., Olmos D., Angel Lorente M., Velaz I., Gonzalez-Benito J. (2018). Preparation and characterization of polymer composite materials based on PLA/TiO_2_ for antibacterial packaging. Polymers.

[67] Buzarovska A., Qualandi C., Parrilli A., Scandola M. (2015). Effect of TiO_2_ nanoparticle loading on poly(L-lactic acid) porous scaffolds fabricated by TIPS. Compos. Part. B. Eng..

[68] Buzarovska A., Dinescu S., Chitoiu L., Costache M. (2018). Porous poly(L-lactic acid) nanocomposite scaffolds with functionalized TiO_2_ nanoparticles: Properties, cytocompatibility and drug release capability. J. Mater. Sci..

[69] Song M., Pan C., Li J.Y., Wang X.M., Gu Z.Z. (2006). Electrochemical study on synergistic effect of the blending of nano TiO_2_ and PLA polymer on the interaction of antitumor drug with DNA. Electroanalysis.

[70] Song M., Pan C., Chen C., Li J.Y., Wang X.M., Gu Z.Z. (2008). The application of new nanocomposites: Enhancement effect of polylactide nanofibers/nano-TiO_2_ blends on biorecognition of anticancer drug daunorubicin. Appl. Surf. Sci..

[71] Shebi A., Lisa S. (2019). Evaluation of biocompatibility and bactericidal activity of hierarchically porous PLA-TiO_2_ nanocomposite films fabricated by breath-figure method. Mater. Chem. Phys..

[72] Lizundia L., Vilas J.L., Sangroniz A., Etxeberria A. (2017). Light and gas barrier properties of PLLA/metallic nanoparticles composite films. Eur. Polym. J..

[73] Wang Z., Pan Z.J., Wang J.G., Zhao R.Z. (2016). A novel hierarchical structured poly(lactic acid)/titania fibrous membrane with excellent antibacterial activity and air filtration performance. J. Nanomater..

[74] Wu W., Liu T., Zhang D., Sun Q., Cao K., Zha J., Lu Y., Wang B., Cao X., Feng Y. (2019). Significantly improved dielectric properties of polylactide nanocomposites via TiO_2_ decorated carbon nanotubes. Comp. Part A Appl. Sci..

[75] Barut N., Shaikh T., Kaur H. (2017). A PLA–TiO_2_ particle brush as a novel support for CuNPs: A catalyst for the fast-sequential reduction and N-arylation of nitroarenes. New J. Chem..

